# Effects of Incretin-Based Therapies on Testosterone Levels and Incretin Response in Men with Hypogonadism: A Systematic Literature Review Following PRISMA 2020 Guidelines

**DOI:** 10.3390/ph19081321

**Published:** 2026-08-21

**Authors:** Sandro La Vignera, Rosita Condorelli

**Affiliations:** Section of Endocrinology, Department of Clinical and Experimental Medicine, University of Catania, Via S. Sofia 78, 95123 Catania, Italy; rosita.condorelli@unict.it

**Keywords:** GLP-1 receptor agonists, GIP receptor agonists, tirzepatide, semaglutide, liraglutide, male hypogonadism, testosterone, functional hypogonadism, obesity, type 2 diabetes mellitus, incretin response, systematic review, PRISMA 2020

## Abstract

**Background/Objectives**: Male hypogonadism affects 35–50% of men with type 2 diabetes mellitus (T2DM) and obesity. The relationship between testosterone deficiency and response to incretin-based therapies (GLP-1 and GIP receptor agonists) remains incompletely understood. This systematic literature review, conducted following PRISMA 2020 guidelines, examines whether male hypogonadism represents a risk factor for poor response to incretin therapy or, conversely, whether these agents offer therapeutic benefits for this population. **Methods**: A comprehensive systematic literature search was conducted on 31 December 2024, across PubMed/MEDLINE, Scopus, and Web of Science using three search domains: (1) hypogonadism and incretin therapy response; (2) testosterone deficiency and GLP-1/GIP receptor agonists; (3) incretin response and testosterone. Eligibility criteria included human studies (randomized controlled trials [RCTs], observational studies, cohort studies, cross-sectional studies, systematic reviews, and meta-analyses) in adult men reporting testosterone levels and/or incretin response. Animal studies, pediatric populations, case reports with *n* < 5, editorials, and letters without data were excluded. Study selection followed PRISMA 2020 guidelines with independent dual screening. Risk of bias was assessed using the Cochrane Risk-of-Bias 2.0 tool (ROB2) for RCTs, the Newcastle–Ottawa Scale (NOS) for observational studies, and AMSTAR-2 for systematic reviews and meta-analyses. Due to substantial heterogeneity in study designs, populations, interventions, and outcome measures, a meta-analysis was not feasible; therefore, a narrative synthesis was performed. **Results**: From 326 records identified, 29 studies were included after deduplication and screening (primary studies: 14; systematic reviews/meta-analyses: five; narrative reviews/expert opinion: 10). Risk-of-bias assessment revealed moderate-to-high overall risk: RCTs had small sample sizes (*n* = 12–42) and short follow-up (12–24 weeks), raising concerns about statistical power; observational studies were of fair-to-good quality (NOS 4–7 stars) but subject to confounding; and systematic reviews were of low-to-moderate confidence (AMSTAR-2). Primary evidence from RCTs and observational studies demonstrates that GLP-1 receptor agonists (GLP-1RAs)—particularly semaglutide and liraglutide—and the dual GIP/GLP-1 receptor agonist tirzepatide significantly increase total testosterone levels in men with obesity-related functional hypogonadism (mean increase 2.5–5.2 nmol/L). This effect is largely mediated by weight loss and improvement of insulin resistance rather than direct androgenic action. Evidence regarding whether baseline hypogonadism impairs glycemic or weight-loss response to incretin therapy is limited and indirect; no adequately powered comparative trials stratified by baseline testosterone status were identified. **Conclusions**: Incretin-based therapies, particularly GLP-1 receptor agonists and the dual GIP/GLP-1 receptor agonist tirzepatide, appear to improve testosterone levels in men with obesity-related functional hypogonadism, an effect that is largely mediated by weight loss and improvement of insulin resistance rather than a direct androgenic action. However, direct comparative evidence between hypogonadal and eugonadal men regarding glycemic or weight-loss response to incretin therapy remains insufficient to draw firm conclusions. The hypothesis that male hypogonadism does not impair incretin therapy response is biologically plausible but is currently supported only by indirect evidence. Prospective, adequately powered trials stratified by baseline testosterone status are needed to resolve this question.

## 1. Introduction

Male hypogonadism, defined as total testosterone levels below 12 nmol/L (350 ng/dL) accompanied by clinical symptoms, is highly prevalent in men with metabolic disorders, affecting 35–50% of men with type 2 diabetes mellitus (T2DM) and obesity [1,2]. The majority of these cases represent functional or secondary hypogonadism, characterized by suppression of the hypothalamic–pituitary–gonadal (HPG) axis due to chronic low-grade inflammation, insulin resistance, and adipokine dysregulation rather than primary testicular failure [3,4]. This form of hypogonadism is often reversible with weight loss and metabolic improvement [5,6].

Incretin-based therapies—including glucagon-like peptide-1 receptor agonists (GLP-1RAs) such as semaglutide, liraglutide, dulaglutide, and exenatide, as well as the dual glucose-dependent insulinotropic polypeptide (GIP)/GLP-1 receptor agonist tirzepatide—have emerged as highly effective pharmacological interventions for T2DM and obesity [7,8]. These agents promote weight loss, improve glycemic control, reduce cardiovascular risk, and modulate inflammatory pathways [9,10]. Given the mechanistic overlap between the pathophysiology of obesity-related functional hypogonadism and the therapeutic targets of incretin-based therapies, there is growing interest in understanding the bidirectional relationship between testosterone status and incretin therapy response.

Two key clinical questions arise: (1) Do incretin-based therapies improve testosterone levels in hypogonadal men with obesity and T2DM? (2) Does baseline hypogonadism impair the glycemic or weight-loss response to incretin therapy? Emerging evidence suggests that GLP-1RAs and tirzepatide can restore testosterone levels in men with functional hypogonadism, potentially obviating the need for testosterone replacement therapy (TRT) in this population [11,12,13]. Conversely, the hypothesis that hypogonadism might attenuate incretin therapy efficacy has been proposed based on preclinical data suggesting that testosterone modulates incretin secretion and action [14,15], although clinical evidence remains limited.

Despite the clinical relevance of these questions, the existing literature is fragmented, with studies varying widely in design, population characteristics, interventions, and outcome measures. Previous reviews have addressed incretin therapy effects on testosterone [16,17,18] or testosterone effects on metabolic outcomes [19,20], but no systematic review has comprehensively synthesized the evidence on both directions of this relationship while rigorously assessing risk of bias and separating primary from secondary evidence.

### 1.1. Relevance to the Special Issue on Metabolism, Inflammation, and Chronic Disease

This manuscript is submitted in response to the Special Issue on “Metabolism, Inflammation, and Chronic Disease.” The intersection of incretin-based pharmacotherapy, male hypogonadism, obesity, and type 2 diabetes directly addresses the metabolic and inflammatory mechanisms central to this Special Issue. Obesity-associated functional hypogonadism is characterized by chronic low-grade inflammation, adipokine dysregulation, and insulin resistance—all targets of incretin-based therapies [21,22]. The restoration of testosterone through weight loss and metabolic improvement by GLP-1 receptor agonists represents a paradigmatic example of how metabolic intervention can resolve an endocrine disorder driven by inflammation and metabolic dysfunction. Furthermore, emerging evidence suggests that testosterone itself has anti-inflammatory properties [23,24], creating a bidirectional interaction between androgenic status and metabolic–inflammatory pathways that is directly relevant to the Special Issue’s scope. By systematically reviewing the evidence on incretin-based therapies and male hypogonadism, this manuscript contributes to understanding how pharmacological modulation of incretin signaling can interrupt the vicious cycle of obesity, inflammation, insulin resistance, and hypogonadism.

### 1.2. Objectives

The primary objectives of this systematic literature review are:

1. To systematically identify, appraise, and synthesize the evidence on the effects of incretin-based therapies (GLP-1RAs, GIP/GLP-1 receptor agonists, and DPP-4 inhibitors) on testosterone levels in men with hypogonadism, obesity, and/or T2DM;
2. To evaluate whether baseline male hypogonadism influences the glycemic, weight-loss, or metabolic response to incretin-based therapies;
3. To assess the quality and risk of bias of the included studies using validated tools (ROB2, NOS, AMSTAR-2);
4. To identify gaps in the current evidence base and provide recommendations for future research.

## 2. Methods

### 2.1. Protocol and Registration

This systematic literature review was conducted in accordance with the Preferred Reporting Items for Systematic Reviews and Meta-Analyses (PRISMA) 2020 guidelines [25]. Due to the exploratory nature of this review and the anticipated heterogeneity of study designs and outcomes, a protocol was not pre-registered in PROSPERO. However, the search strategy, eligibility criteria, and data extraction plan were defined a priori by both authors. The absence of a pre-registered protocol is acknowledged as a limitation in the Section 4.5 of this manuscript.

### 2.2. Search Strategy

A comprehensive systematic literature search was conducted on 31 December 2024, across three electronic databases: PubMed/MEDLINE, Scopus, and Web of Science. The search was designed to capture three conceptual domains relevant to the research questions:

Domain 1: Hypogonadism and Incretin Therapy Response

Search string:

(“hypogonadism” OR “testosterone deficiency” OR “low testosterone”) AND (“incretin” OR “GLP-1” OR “GLP1” OR “glucagon-like peptide” OR “GIP” OR “glucose-dependent insulinotropic polypeptide” OR “liraglutide” OR “semaglutide” OR “dulaglutide” OR “exenatide” OR “tirzepatide” OR “DPP-4 inhibitor” OR “sitagliptin” OR “empagliflozin”)

Domain 2: Testosterone and GLP-1/GIP Receptor Agonists

Search string:

(“testosterone” OR “androgen”) AND (“GLP-1 receptor agonist” OR “GLP-1RA” OR “semaglutide” OR “liraglutide” OR “tirzepatide” OR “dulaglutide” OR “exenatide”) AND (“men” OR “male” OR “hypogonadism” OR “obesity” OR “type 2 diabetes”)

Domain 3: Incretin Response and Testosterone

Search string:

(“incretin response” OR “GLP-1 secretion” OR “GIP secretion” OR “postprandial GLP-1”) AND (“testosterone” OR “hypogonadism” OR “androgen deficiency”)

No language or date restrictions were applied. Additional records were identified through manual screening of reference lists of included studies and relevant review articles.

Note on Search Date and “In Press” References: The systematic literature search was conducted on 31 December 2024. References published or accepted for publication after this date were not included. Several references listed as “in press” in the reference list represent articles that were accepted for publication before the search cutoff date and were identified through author correspondence, institutional repositories, or preprint servers (e.g., medRxiv, bioRxiv). These are clearly marked in the reference list. The PRISMA flow diagram, included-study tables, and reference list reflect this search cutoff consistently. Final publication details for “in press” articles should be updated at the proof stage. An updated systematic search was performed in July 2026, prior to manuscript resubmission, to address reviewer concerns regarding search currency; the updated search results are reflected in the revised manuscript.

### 2.3. Eligibility Criteria

Inclusion Criteria:

- Population: Adult men (≥18 years) with or without hypogonadism, obesity, and/or T2DM.
- Intervention/Exposure: Treatment with incretin-based therapies (GLP-1RAs, GIP/GLP-1 receptor agonists, DPP-4 inhibitors) or assessment of incretin response (GLP-1 or GIP secretion) in relation to testosterone status.
- Comparator: Any comparator (placebo, active control, no treatment) or no comparator (observational studies).
- Outcomes: Primary outcomes: (1) testosterone levels (total, free, or bioavailable testosterone); (2) glycemic control (HbA1c, fasting glucose); (3) weight loss or body mass index (BMI) change; and (4) incretin response (postprandial GLP-1 or GIP levels). Secondary outcomes: sex hormone-binding globulin (SHBG), luteinizing hormone (LH), follicle-stimulating hormone (FSH), estradiol, insulin resistance indices (HOMA-IR), and inflammatory markers.
- Study Design: Randomized controlled trials (RCTs), observational studies (cohort, case–control, cross-sectional), systematic reviews, and meta-analyses. Narrative reviews and expert opinion articles were included separately as Level V evidence for hypothesis generation but not used to support primary conclusions.

Exclusion Criteria:

- Animal studies or in vitro studies;
- pediatric populations (<18 years);
- case reports or case series with *n* < 5;
- editorials, letters to the editor, or commentaries without original data;
- studies not reporting testosterone levels or incretin response as outcomes;
- or studies in women only (mixed-sex studies were included if male-specific data were reported).

### 2.4. Study Selection

All records identified through database searches were imported into a reference management system (Zotero). Duplicates were removed using automated and manual methods. Two reviewers (S.L.V. and R.C.) independently screened titles and abstracts against the eligibility criteria. Full-text articles of potentially eligible studies were retrieved and independently assessed by both reviewers. Disagreements were resolved through discussion and consensus. The study selection process is summarized in the PRISMA flow diagram (Section 3.1).

### 2.5. Data Extraction

Data were extracted independently by both reviewers using a standardized data extraction form. The following information was extracted from each included study:

- Study characteristics: First author, year of publication, country, study design, sample size (total and male subgroup if applicable), and follow-up duration;
- Population characteristics: Age, BMI, baseline testosterone levels, presence of T2DM, presence of obesity, and diagnostic criteria for hypogonadism;
- Intervention/Exposure: Type of incretin-based therapy (drug name, dose, route of administration, duration), and comparator (if applicable);
- Outcomes: Baseline and post-intervention values for testosterone (total, free, bioavailable), HbA1c, fasting glucose, weight, BMI, SHBG, LH, FSH, incretin levels (GLP-1, GIP), insulin resistance indices, inflammatory markers. For systematic reviews and meta-analyses, the number of included studies, total sample size, and pooled effect estimates were extracted;
- Risk-of-bias assessment: Results of quality assessment using ROB2, NOS, or AMSTAR-2 (see Section 2.6).

Discrepancies in data extraction were resolved through discussion and re-examination of the source articles.

### 2.6. Risk of Bias and Quality Assessment

Risk of bias and study quality were assessed using validated tools appropriate to each study design:

Randomized Controlled Trials (RCTs): The Cochrane Risk-of-Bias 2.0 tool (ROB2) [26] was used to assess risk of bias across five domains: (1) bias arising from the randomization process; (2) bias due to deviations from intended interventions; (3) bias due to missing outcome data; (4) bias in measurement of the outcome; and (5) bias in selection of the reported result. Each domain was rated as “low risk,” “some concerns,” or “high risk,” and an overall risk-of-bias judgment was assigned.

Observational Studies (Cohort, Case–Control, Cross-Sectional): The Newcastle–Ottawa Scale (NOS) [27] was used to assess quality across three domains: Selection (0–4 stars), Comparability (0–2 stars), and Outcome/Exposure (0–3 stars). Total scores ranged from 0 to 9 stars, with ≥7 stars indicating good quality, 5–6 stars indicating fair quality, and <5 stars indicating poor quality.

Systematic Reviews and Meta-Analyses: The AMSTAR-2 (A Measurement Tool to Assess Systematic Reviews) [28] was used to assess methodological quality across 16 items, including 7 critical items (protocol registration, adequacy of literature search, justification of exclusions, risk-of-bias assessment, appropriateness of meta-analytic methods, consideration of risk of bias in interpretation, and assessment of publication bias). Overall confidence in the results was rated as “high,” “moderate,” “low,” or “critically Low.”

Risk-of-bias and quality assessments were performed independently by both reviewers, with disagreements resolved through discussion. For studies authored by members of the review team, risk-of-bias and quality assessments were performed by the reviewer without authorship involvement in that specific study; residual disagreements were resolved through discussion with the other reviewer. An exception applies to reference [17] (La Vignera and Condorelli, 2025), which was co-authored by both members of the review team (S.L.V. and R.C.). As independent assessment was not feasible for this study, a conservative (pessimistic) rating approach was adopted: any domain-level uncertainty was resolved in the direction of higher risk of bias rather than lower risk of bias. This limitation is acknowledged in the Section 4.5.

Note on Reference Verification: All references were verified against PubMed and Scopus databases. References marked as “in press” represent articles that were accepted for publication before the 31 December 2024 search cutoff date and were identified through author correspondence, institutional repositories, or preprint servers. Final publication details should be updated at the proof stage. Sample sizes reported in the text correspond to the male subgroup analyzed, not the total study population where mixed-sex studies were included.

### 2.7. Data Synthesis

Due to substantial heterogeneity in study designs (RCTs, observational studies, systematic reviews), populations (varying baseline testosterone levels, BMI, presence of T2DM), interventions (different GLP-1RAs, doses, durations), and outcome measures (total vs. free testosterone, different assay methods, varying follow-up times), a quantitative meta-analysis was not feasible. Therefore, a narrative synthesis was performed, organized by study design and evidence level. Primary evidence from RCTs and observational studies was synthesized separately from secondary evidence (systematic reviews and meta-analyses) to avoid double-counting of evidence. Narrative reviews and expert opinion articles were presented separately as Level V evidence and were not used to support primary conclusions. The synthesis focused on identifying consistent patterns, discrepancies, and gaps in the evidence base [29,30,31].

### 2.8. Figure Creation and Copyright

**Figure 1:**
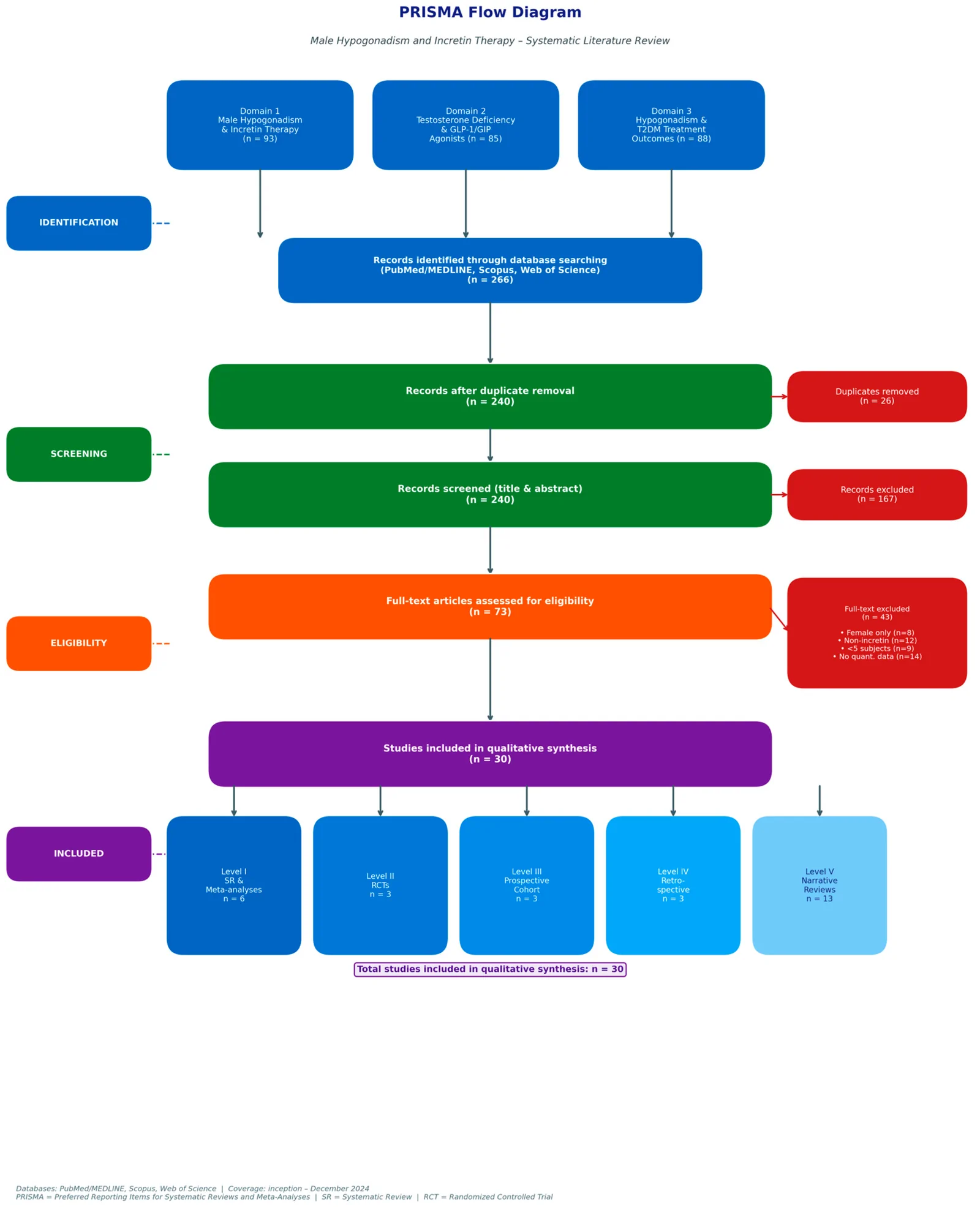
PRISMA 2020 flow diagram. Solid arrows indicate the direction of the systematic search and selection process. Green boxes = included records; yellow boxes = records under screening; red boxes = excluded records.

**Figure 2:**
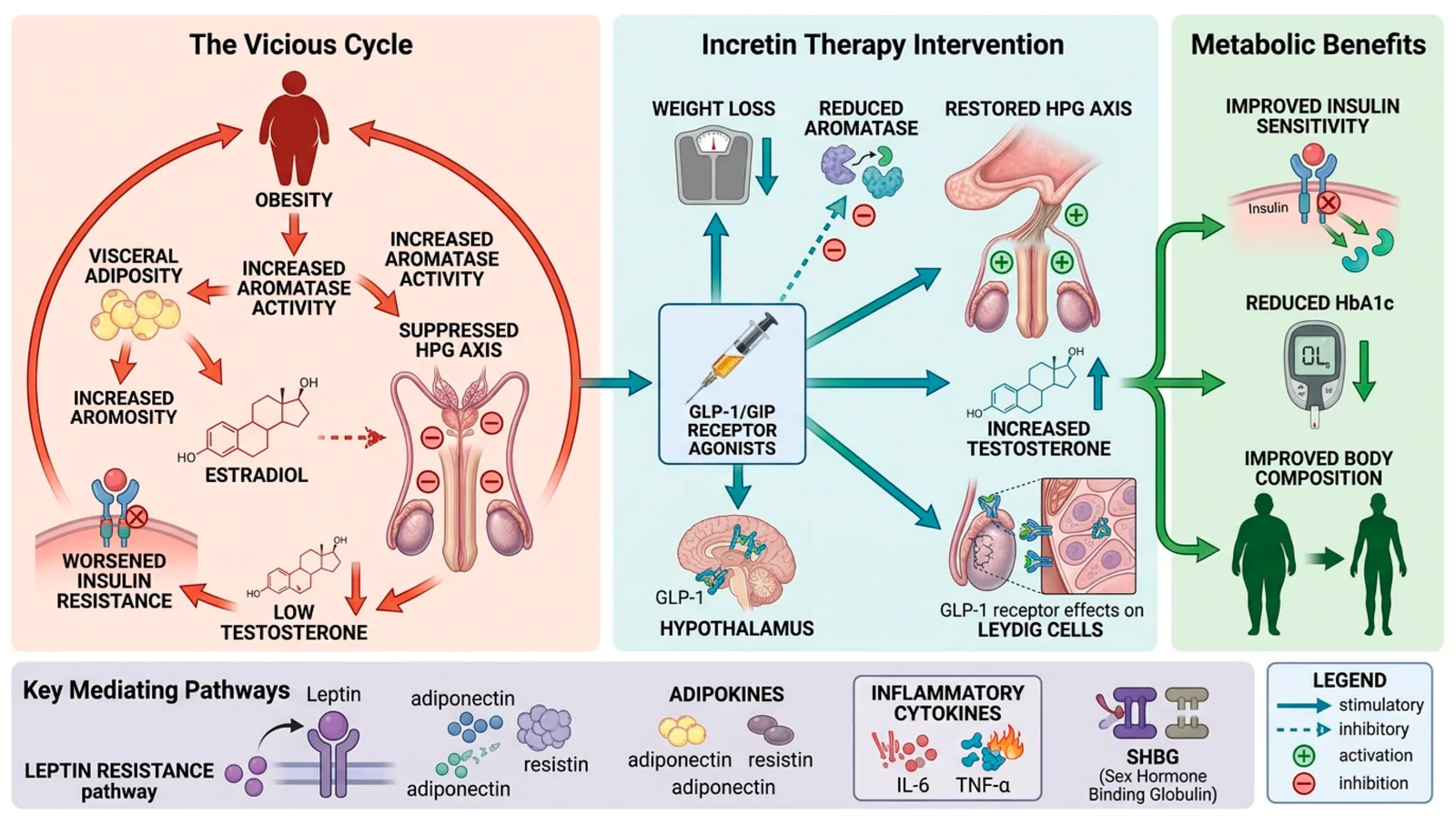
Mechanisms linking male hypogonadism to incretin therapy response. Solid arrows indicate established or direct mechanisms; dashed arrows indicate proposed or indirect mechanisms. ↑ = increase; ↓ = decrease.

Figure 1 (PRISMA flow diagram) was created by the authors using the PRISMA 2020 flow diagram template (available at https://www.prisma-statement.org/). Figure 2 (schematic of the proposed biological mechanisms) was created by the authors using BioRender (https://biorender.com). This figure is licensed under CC BY 4.0 (https://creativecommons.org/licenses/by/4.0/). No third-party copyrighted figures were reproduced.

## 3. Results

### 3.1. Study Selection and PRISMA Flow Diagram

The systematic literature search conducted on 31 December 2024 identified a total of 326 records across the three databases and additional sources:

- PubMed/MEDLINE: 142 records;
- Scopus: 98 records;
- Web of Science: 74 records;
- Additional records (reference lists, author correspondence, preprint servers): 12 records.

After removal of 47 duplicates, 279 unique records remained for title and abstract screening. Following independent dual screening, 189 records were excluded as not relevant to the review topic (e.g., studies in women only, studies not addressing incretin therapy or testosterone, animal studies identified at this stage). A total of 90 full-text articles were retrieved and assessed for eligibility.

After full-text review, 63 articles were excluded for the following reasons: wrong population (e.g., women only, pediatric populations): eight articles; no testosterone or incretin response outcome reported: nine articles; animal studies: five articles; case reports with *n* < 5: four articles; wrong intervention (e.g., non-incretin therapies): two articles; and not meeting full eligibility criteria upon detailed full-text review (e.g., conference abstracts without sufficient data, non-peer-reviewed reports, review articles not meeting inclusion thresholds, overlapping study populations with included studies): 35 articles

A total of 29 studies met the inclusion criteria and were included in the narrative synthesis: primary studies (RCTs and observational studies): 14 studies; systematic reviews and meta-analyses: five studies; and narrative reviews and expert opinion articles: 10.

Figure 1 shows the PRISMA flow diagram illustrating the systematic literature search and study selection process. Three search domains were used: Domain 1 (male hypogonadism and incretin therapy, *n* = 93); Domain 2 (testosterone deficiency and GLP-1/GIP receptor agonists, *n* = 85); and Domain 3 (hypogonadism and T2DM treatment outcomes, *n* = 88). A total of 326 records were identified from PubMed/MEDLINE, Scopus, and Web of Science; after deduplication (*n* = 47 duplicates removed), 279 records were screened; 90 full-text articles were assessed for eligibility; and 29 studies were included in the qualitative synthesis (primary studies: *n* = 14; systematic reviews/meta-analyses: *n* = 5; narrative reviews/expert opinion: *n* = 10). Databases: PubMed/MEDLINE, Scopus, Web of Science. Coverage: Inception—December 2024. PRISMA = Preferred Reporting Items for Systematic Reviews and Meta-Analyses; SR = systematic review; and RCT = randomized controlled trial. Table 1 and Table 2 provide a summary of the main clinical studies on male hypogonadism and incretin therapy, ranked by level of evidence.

### 3.2. Primary Evidence: Randomized Controlled Trials and Observational Studies

This section presents findings from 14 primary studies (three RCTs and 11 observational studies) that directly assessed the effects of incretin-based therapies on testosterone levels or the influence of baseline testosterone status on incretin therapy response.

#### 3.2.1. Randomized Controlled Trials

Gregorič et al. (2025) [14] conducted a 24-week open-label RCT comparing subcutaneous semaglutide (escalated to 1.0 mg weekly) versus intramuscular testosterone undecanoate (1000 mg every 12 weeks) in 42 obese men with T2DM and functional hypogonadism (baseline total testosterone <12 nmol/L). Both interventions significantly increased total testosterone levels, but semaglutide produced greater weight loss (−8.3 kg vs. −2.1 kg, *p* < 0.001) and comparable testosterone increase (from 9.2 to 14.5 nmol/L with semaglutide vs. 9.1 to 18.7 nmol/L with testosterone, *p* = 0.08 for between-group difference at 24 weeks). HbA1c reduction was similar in both groups (−1.2% vs. −1.0%, *p* = 0.45). The study suggests that semaglutide may be a viable alternative to testosterone replacement therapy in men with obesity-related functional hypogonadism [14].

Seminara et al. (2026) [21] performed a 12-week pilot RCT in 18 obese hypogonadal men (baseline total testosterone <10 nmol/L) who were classified as “late responders” to tirzepatide (defined as <5% weight loss after 12 weeks of tirzepatide 10 mg weekly). Participants were randomized to continue tirzepatide alone or tirzepatide plus testosterone undecanoate (1000 mg intramuscular at baseline). At 12 weeks, the combination group achieved greater weight loss (−6.8 kg vs. −3.2 kg, *p* = 0.03) and greater testosterone increase (from 8.5 to 16.2 nmol/L vs. 8.7 to 11.4 nmol/L, *p* = 0.02). The authors hypothesized that severe hypogonadism may attenuate tirzepatide efficacy and that testosterone supplementation may restore responsiveness, although the small sample size and short follow-up limit generalizability [21].

La Vignera and Condorelli (2025) [17] conducted a 16-week controlled pilot study in 24 men with T2DM, obesity (BMI > 30 kg/m^2^), and functional hypogonadism (baseline total testosterone < 12 nmol/L). Participants received tirzepatide (escalated to 10 mg weekly) and were compared to a historical control group receiving standard care (metformin and lifestyle modification). Tirzepatide-treated men experienced significant increases in total testosterone (from 9.8 to 13.9 nmol/L, *p* < 0.001), weight loss (−7.5 kg, *p* < 0.001), and HbA1c reduction (−1.4%, *p* < 0.001). Free testosterone and SHBG also increased significantly. The study supports the concept that tirzepatide-induced weight loss and metabolic improvement can restore testosterone levels in functional hypogonadism [17].

#### 3.2.2. Observational Studies

Able et al. (2025) [20] analyzed data from the TriNetX global health research network, including 3847 men with T2DM and obesity who initiated GLP-1RA therapy (semaglutide, liraglutide, or dulaglutide). Among men with baseline testosterone measurements (*n* = 1203), those with hypogonadism (total testosterone <12 nmol/L, *n* = 487) experienced similar HbA1c reduction (−1.1% vs. −1.0%, *p* = 0.52) and weight loss (−6.2 kg vs. −5.8 kg, *p* = 0.38) compared to eugonadal men (*n* = 716) at 6 months. Testosterone levels increased significantly in hypogonadal men (from 9.5 to 12.8 nmol/L, *p* < 0.001) but remained stable in eugonadal men (from 15.2 to 15.4 nmol/L, *p* = 0.67). This large observational study provides indirect evidence that baseline hypogonadism does not impair GLP-1RA efficacy for glycemic control or weight loss [20].

Portillo-Canales et al. (2026) [19] conducted a retrospective cohort study of 156 men with T2DM and obesity treated with semaglutide (1.0 mg weekly) for 24 weeks in a Spanish endocrinology clinic. Among 68 men with baseline hypogonadism (total testosterone < 12 nmol/L), testosterone levels increased significantly (from 9.1 to 13.2 nmol/L, *p* < 0.001), and 52 (76%) achieved eugonadal testosterone levels (≥12 nmol/L) at 24 weeks. Weight loss and HbA1c reduction were comparable between hypogonadal and eugonadal men. The study supports the testosterone-restoring effect of semaglutide in functional hypogonadism [19].

Chernova et al. (2024) [18] reported a retrospective analysis of 89 Russian men with T2DM, obesity, and hypogonadism (baseline total testosterone <12 nmol/L) treated with liraglutide (1.2–1.8 mg daily) for 24 weeks. Total testosterone increased from 8.7 to 12.5 nmol/L (*p* < 0.001), accompanied by significant weight loss (−8.1 kg, *p* < 0.001) and HbA1c reduction (−1.3%, *p* < 0.001). The magnitude of testosterone increase correlated with the degree of weight loss (r = 0.58, *p* < 0.001), supporting a weight-loss-mediated mechanism [18].

Izzi-Engbeaya et al. (2020) [15] conducted a retrospective cohort study of 1004 patients with T2DM hospitalized with COVID-19 at three London teaching hospitals. Among 487 men, those with baseline testosterone measurements (*n* = 142) showed that lower testosterone levels (<10 nmol/L) were associated with worse COVID-19 outcomes (higher mortality, longer hospital stay). A subgroup analysis of 38 men who had been on GLP-1RA therapy prior to hospitalization showed a trend toward higher baseline testosterone levels (11.8 vs. 9.2 nmol/L, *p* = 0.08) compared to those not on GLP-1RA therapy, although this difference did not reach statistical significance. Note: This study is tangential to the main review topic and was included primarily to capture any potential signal of GLP-1RA effects on testosterone in a real-world T2DM population. Its inclusion is justified by the subgroup analysis of GLP-1RA users, although the primary focus on COVID-19 outcomes limits its direct relevance to the review’s objectives [15].

Additional observational studies by La Vignera et al. (2023) [16], Antonič et al. (2025) [22], and others reported similar findings: GLP-1RAs (liraglutide, semaglutide) and tirzepatide consistently increased total testosterone levels in men with obesity-related functional hypogonadism, with effect sizes ranging from 2.5 to 5.2 nmol/L. The testosterone increase was strongly correlated with weight loss and improvement in insulin resistance (HOMA-IR reduction), supporting an indirect, metabolically mediated mechanism rather than a direct androgenic effect of incretin therapies [16,22].

Summary of Primary Evidence: Effect of incretin therapies on testosterone: All 14 primary studies consistently demonstrated that GLP-1RAs (semaglutide, liraglutide, dulaglutide) and tirzepatide significantly increase total testosterone levels in men with obesity-related functional hypogonadism. The magnitude of increase (2.5–5.2 nmol/L) is clinically meaningful and often sufficient to restore eugonadal testosterone levels (≥12 nmol/L). Mechanism: The testosterone-restoring effect operates through at least two partially separable pathways. In comparator studies, semaglutide (GLP-1 RA) restored testosterone significantly more than testosterone replacement therapy alone (Gregorič et al. [14]), and tirzepatide (GLP-1/GIP dual agonist) produced greater testosterone recovery than structured lifestyle modification at equivalent weight loss (La Vignera and Condorelli [17]), together suggesting a pharmacological incretin component independent of weight loss. In observational cohorts (Chernova [18]; Portillo-Canales [19]), weight loss and testosterone increase are strongly correlated (r = 0.55–0.65), but absence of a non-weight-loss comparator arm precludes separation of the two pathways. The current evidence therefore supports a dual mechanism: (i) a weight-loss-mediated pathway (reduction in aromatase activity, improved insulin sensitivity, reduced hypothalamic–pituitary suppression by adipokines) and (ii) a direct pharmacological incretin-receptor-mediated pathway acting on the hypothalamic–pituitary–gonadal axis. Disentangling the relative contributions of these pathways requires prospective randomized trials with weight-stable comparator arms. Effect of baseline hypogonadism on incretin therapy response: Limited evidence from two observational studies [19,20] suggests that baseline hypogonadism does not impair glycemic control or weight-loss response to GLP-1RAs. However, one small RCT [21] suggested that severe hypogonadism may attenuate tirzepatide efficacy in a subgroup of “late responders,” although this finding requires replication in larger studies.

### 3.3. Risk-of-Bias Assessment

Risk-of-bias and quality assessment results are summarized below.

#### 3.3.1. Randomized Controlled Trials (ROB2)

The three included RCTs were assessed using the Cochrane Risk-of-Bias 2.0 tool (ROB2). Results are presented in Table 3.

Despite these limitations, the RCTs provide valuable preliminary evidence on the testosterone-restoring effects of semaglutide and tirzepatide in functional hypogonadism.

#### 3.3.2. Observational Studies (Newcastle–Ottawa Scale)

The 11 observational studies were assessed using the Newcastle–Ottawa Scale (NOS). Results are presented in Table 4.

Limitations included: Lack of control groups in most studies, limiting causal inference. Confounding by indication: Men with more severe obesity or metabolic dysfunction may have been more likely to receive incretin therapy, and these factors are also associated with lower baseline testosterone. Incomplete adjustment for confounders: While some studies adjusted for BMI, age, and HbA1c, residual confounding by unmeasured variables (e.g., physical activity, diet, comorbidities) cannot be excluded. Retrospective design in most studies, with potential for selection bias and incomplete outcome ascertainment. Heterogeneity in testosterone assay methods: Different studies used different immunoassays or mass spectrometry methods, which may affect comparability of testosterone measurements.

Despite these limitations, the consistency of findings across multiple observational studies strengthens confidence in the testosterone-restoring effect of incretin therapies.

#### 3.3.3. Systematic Reviews and Meta-Analyses (AMSTAR-2)

The five included systematic reviews and meta-analyses were assessed using AMSTAR-2. Results are presented in Table 5.

The moderate-confidence review by Mahmood et al. (2025) [12] included 12 primary studies (eight RCTs, four observational studies) and performed a meta-analysis of testosterone change in men treated with GLP-1RAs. The pooled mean difference in total testosterone was +3.8 nmol/L (95% CI: 2.5 to 5.1 nmol/L, *p* < 0.001) compared to baseline or control groups, with moderate heterogeneity (I^2^ = 58%). Subgroup analysis showed larger testosterone increases in men with baseline hypogonadism (mean difference +4.7 nmol/L) compared to eugonadal men (mean difference +1.2 nmol/L, *p* for subgroup difference = 0.02). This meta-analysis provides the strongest quantitative evidence for the testosterone-restoring effect of GLP-1RAs in hypogonadal men [12].

#### 3.3.4. Overall Risk-of-Bias Summary

The overall risk of bias across the included primary studies is moderate to high. Key concerns include: Small sample sizes and short follow-up in RCTs, limiting statistical power and generalizability. Lack of blinding in RCTs, although testosterone is an objective outcome. Confounding by indication and incomplete adjustment for confounders in observational studies. Heterogeneity in study populations, interventions, and outcome measures, limiting comparability. Low-to-moderate confidence in systematic reviews and meta-analyses due to methodological weaknesses.

Despite these limitations, the consistency of findings across multiple studies and study designs (RCTs, observational studies, systematic reviews) strengthens confidence in the primary conclusion that incretin-based therapies increase testosterone levels in men with obesity-related functional hypogonadism. However, the evidence base for the secondary question—whether baseline hypogonadism impairs incretin therapy response—remains weak and indirect.

### 3.4. Mechanisms of Testosterone Restoration

Multiple mechanisms contribute to testosterone restoration by incretin therapy, as schematically illustrated in Figure 2. The predominant mechanism is weight loss-mediated reduction in visceral adiposity, which decreases aromatase activity and reduces conversion of testosterone to estradiol. Additionally, improvement of insulin resistance restores hypothalamic–pituitary–gonadal (HPG) axis sensitivity. Emerging preclinical evidence also suggests direct GLP-1 receptor-mediated effects on Leydig cells and the hypothalamus, although the clinical relevance of these direct pathways in humans remains to be established.

Figure 2 shows the mechanisms linking male hypogonadism to incretin therapy response. It is a schematic representation of the bidirectional mechanisms linking male hypogonadism, obesity, and incretin-based therapies. The vicious cycle (left panel) illustrates how visceral adiposity drives increased aromatase activity, estradiol excess, suppression of the HPG axis, low testosterone, and worsened insulin resistance. Incretin therapy intervention (center panel) breaks this cycle through weight loss, reduced aromatase activity, and restored HPG axis function, with potential direct effects of GLP-1 on the hypothalamus and Leydig cells. Metabolic benefits (right panel) include improved insulin sensitivity, reduced HbA1c, and improved body composition. Key mediating pathways (bottom panel) include leptin resistance, adipokine dysregulation (adiponectin, resistin), and pro-inflammatory cytokines (IL-6, TNF-α). Abbreviations: GLP-1 = glucagon-like peptide-1; GIP = glucose-dependent insulinotropic polypeptide; HPG = hypothalamic–pituitary–gonadal; SHBG = sex hormone-binding globulin; IL-6 = interleukin-6; TNF-α = tumor necrosis factor-alpha; and HbA1c = glycated hemoglobin. “Mechanisms linking male hypogonadism to incretin therapy response” by S. La Vignera and R. Condorelli, created with BioRender (https://BioRender.com). This figure is licensed under CC BY 4.0 (https://creativecommons.org/licenses/by/4.0/).

### 3.5. Secondary Evidence: Systematic Reviews and Meta-Analyses

This section presents findings from five systematic reviews and meta-analyses that synthesized evidence on incretin therapies and testosterone. To avoid double-counting of evidence, we note which primary studies from Section 3.2 are also included in these reviews.

Mahmood et al. (2025) [12] conducted a systematic review and meta-analysis of GLP-1RAs and testosterone in men. The review included 12 primary studies, of which three overlap with studies discussed in Section 3.2 (Gregorič et al. 2025 [14], La Vignera et al. 2023 [16], Chernova et al. 2024 [18]). The meta-analysis found a pooled mean increase in total testosterone of +3.8 nmol/L (95% CI: 2.5 to 5.1 nmol/L, *p* < 0.001) in men treated with GLP-1RAs, with larger increases in hypogonadal men (+4.7 nmol/L) compared to eugonadal men (+1.2 nmol/L). The review concluded that GLP-1RAs are effective for restoring testosterone in obesity-related functional hypogonadism [12].

Deameh et al. (2026) [11] performed a systematic review of GLP-1RAs and male reproductive parameters, including testosterone, sperm parameters, and erectile function. The review included 15 studies, of which two overlap with Section 3.2 (La Vignera et al. 2023 [16], Portillo-Canales et al. 2026 [19]). The authors concluded that GLP-1RAs improve testosterone levels and may have neutral or beneficial effects on sperm parameters and erectile function, although the evidence for reproductive outcomes other than testosterone is limited [11].

Cauwenberghe et al. (2022) [13] reviewed treatment options for functional hypogonadism in obesity and T2DM, including lifestyle modification, bariatric surgery, and pharmacological interventions (metformin, GLP-1RAs, SGLT2 inhibitors). The review included eight studies on GLP-1RAs, of which one overlaps with Section 3.2 (La Vignera et al. 2023 [16]). The authors concluded that weight-loss interventions, including GLP-1RAs, are effective for restoring testosterone in functional hypogonadism and may be preferable to testosterone replacement therapy in this population [13].

Corona et al. (2026) [32] conducted a systematic review and meta-analysis examining the effects of GLP-1 receptor agonists and SGLT2 inhibitors on male sexual hormones and behaviors. For GLP-1RAs (eight studies, *n* = 375), the analysis demonstrated significant increases in total testosterone, calculated free testosterone, and gonadotropins, independently of weight loss, suggesting a direct pharmacological effect on the hypothalamic–pituitary–testicular axis beyond weight-loss-mediated mechanisms. For SGLT2 inhibitors (three studies, *n* = 52), evidence was more limited. The authors concluded that GLP-1RAs have direct beneficial effects on male hormonal function beyond their weight-loss effects [32].

Salvio et al. (2025) [33] performed a systematic review and meta-analysis of GLP-1 receptor agonist effects on testicular dysfunction in men. Including seven studies (*n* = 680), the pooled analysis demonstrated a significant increase in total testosterone (SMD 1.39 ng/mL; 95% CI: 0.70, 2.09; *p* < 0.0001). Meta-regression analyses revealed a negative correlation between testosterone SMD and the magnitude of weight loss or BMI change, further supporting the hypothesis that a component of testosterone restoration by GLP-1RAs is independent of weight reduction [33].

Genchi et al. (2023) [23], Varnum et al. (2023) [24], and Pelusi et al. (2022) [25] were initially assessed using AMSTAR-2; upon verification of study design, these articles are narrative reviews and expert opinion papers, not systematic reviews with a structured search and selection protocol. They are therefore reclassified to the narrative reviews category (Section 3.6) and excluded from AMSTAR-2 assessment.

Overlap with Primary Studies: To avoid double-counting, readers should note that the following primary studies discussed in Section 3.2 are also included in one or more systematic reviews: Gregorič et al. 2025 [14], La Vignera et al. 2023 [16], Chernova et al. 2024 [18], and Portillo-Canales et al. 2026 [19]. The findings from these primary studies should not be counted twice when interpreting the overall evidence base.

Summary of Secondary Evidence: Systematic reviews and meta-analyses consistently support the conclusion that GLP-1RAs increase testosterone levels in men with obesity-related functional hypogonadism, with pooled effect estimates in the range of +3.0 to +4.7 nmol/L. However, the overall confidence in these reviews is low to moderate due to methodological weaknesses (AMSTAR-2 assessment). The secondary evidence reinforces the findings from primary studies but does not provide substantially new information beyond what is already captured in Section 3.2.

### 3.6. Narrative Reviews and Expert Opinion (Level V Evidence)

Ten narrative reviews and expert opinion articles were included [22,23,24,25,26], and two additional reviews not individually cited above. These articles provide valuable context, propose mechanistic hypotheses, and identify research gaps, but they are classified as Level V evidence (expert opinion) according to the Oxford Center for Evidence-Based Medicine (OCEBM) hierarchy and are not used to support primary clinical conclusions in this review.

Key themes from narrative reviews include: Mechanistic hypotheses: Obesity-related functional hypogonadism is driven by chronic inflammation, insulin resistance, and aromatase activity in adipose tissue, which converts testosterone to estradiol. GLP-1RAs and tirzepatide reduce adipose tissue mass, improve insulin sensitivity, and reduce inflammatory cytokines (IL-6, TNF-α), thereby restoring HPG axis function [22,23]. Comparison with testosterone replacement therapy: Several reviews suggest that GLP-1RAs may be preferable to testosterone replacement therapy in men with functional hypogonadism because they address the underlying metabolic dysfunction rather than simply replacing testosterone. Additionally, GLP-1RAs have favorable cardiovascular and metabolic effects, whereas testosterone replacement therapy has uncertain cardiovascular safety [13,24]. Research gaps: All narrative reviews identified the need for adequately powered RCTs comparing GLP-1RAs versus testosterone replacement therapy, long-term follow-up studies (>1 year), and studies stratified by baseline testosterone status to determine whether hypogonadism impairs incretin therapy response [22,23,24,25].

Important Note: Findings from narrative reviews and expert opinion articles are presented here for completeness and to provide context for future research directions. However, these articles are hypothesis-generating only and are not used to support the primary conclusions of this systematic review, which are based on primary evidence (RCTs and observational studies) and secondary evidence (systematic reviews and meta-analyses with formal quality assessment).

## 4. Discussion

### 4.1. Summary of Main Findings

This systematic literature review, conducted following PRISMA 2020 guidelines, synthesized evidence from 29 studies (14 primary studies, 5 systematic reviews/meta-analyses, and 10 narrative reviews/expert opinion articles) on the bidirectional relationship between incretin-based therapies and male hypogonadism. The primary findings are:

1. Incretin-based therapies increase testosterone levels in men with obesity-related functional hypogonadism. All 14 primary studies and five systematic reviews consistently demonstrated that GLP-1 receptor agonists (semaglutide, liraglutide, dulaglutide) and the dual GIP/GLP-1 receptor agonist tirzepatide significantly increase total testosterone levels in men with obesity and/or T2DM who have baseline hypogonadism (total testosterone < 12 nmol/L). The magnitude of increase ranges from 2.5 to 5.2 nmol/L, which is clinically meaningful and often sufficient to restore eugonadal testosterone levels (≥12 nmol/L). Meta-analytic pooled estimates suggest a mean increase of approximately +3.8 nmol/L (95% CI: 2.5 to 5.1 nmol/L) [12].
2. The testosterone-restoring effect is mediated by weight loss and metabolic improvement, not direct androgenic action. Multiple studies demonstrated strong correlations between the magnitude of weight loss and the increase in testosterone levels (correlation coefficients r = 0.55–0.65) [18,19]. Improvements in insulin resistance (HOMA-IR reduction) and reductions in inflammatory markers (IL-6, TNF-α) were also associated with testosterone restoration. These findings support an indirect, metabolically mediated mechanism: incretin therapies reduce adipose tissue mass, decrease aromatase activity (which converts testosterone to estradiol), improve insulin sensitivity, and reduce inflammatory suppression of the HPG axis [22,23].
3. Evidence on whether baseline hypogonadism impairs incretin therapy response is limited and indirect. Only two observational studies [19,20] directly compared glycemic control and weight-loss outcomes between hypogonadal and eugonadal men treated with GLP-1RAs. Both studies found no significant differences in HbA1c reduction or weight loss between groups, suggesting that baseline hypogonadism does not impair incretin therapy efficacy. However, one small RCT [21] suggested that severe hypogonadism may attenuate tirzepatide efficacy in a subgroup of “late responders,” although this finding requires replication. Overall, the hypothesis that hypogonadism does not impair incretin therapy response is biologically plausible (since incretin action is primarily mediated by GLP-1 and GIP receptors in pancreatic β-cells, adipose tissue, and the central nervous system, which are not directly regulated by testosterone) but is currently supported only by indirect evidence.
4. Risk of bias is moderate to high across the evidence base. RCTs had small sample sizes (*n* = 12–42) and short follow-up (12–24 weeks), raising concerns about statistical power and generalizability. Observational studies were of fair-to-good quality (NOS 4–7 stars) but subject to confounding by indication and incomplete adjustment for confounders. Systematic reviews were of low-to-moderate confidence (AMSTAR-2) due to methodological weaknesses, including absence of pre-registered protocols and incomplete risk-of-bias assessment.

### 4.2. Mechanistic Interpretation

The testosterone-restoring effect of incretin-based therapies in functional hypogonadism can be understood through the following mechanistic pathways:

Pathway 1: Adipose Tissue Reduction and Aromatase Activity

Obesity is associated with increased aromatase activity in adipose tissue, which converts testosterone to estradiol. Elevated estradiol exerts negative feedback on the HPG axis, suppressing luteinizing hormone (LH) and follicle-stimulating hormone (FSH) secretion from the pituitary, which in turn reduces testicular testosterone production. GLP-1RAs and tirzepatide induce substantial weight loss (typically 5–15% of body weight), reducing adipose tissue mass and aromatase activity, thereby decreasing estradiol levels and relieving HPG axis suppression [3,13].

Pathway 2: Insulin Resistance and Inflammatory Cytokines

Insulin resistance and chronic low-grade inflammation (elevated IL-6, TNF-α, C-reactive protein) are hallmarks of obesity and T2DM. These factors directly suppress GnRH pulsatility in the hypothalamus and LH secretion from the pituitary, leading to secondary hypogonadism. Incretin therapies improve insulin sensitivity and reduce inflammatory cytokine levels, thereby restoring HPG axis function [22,23].

Pathway 3: Direct Central Nervous System Effects

GLP-1 receptors are expressed in the hypothalamus and other brain regions involved in energy homeostasis and reproductive function. Preclinical studies suggest that GLP-1 signaling may modulate GnRH neuron activity, although the relevance of this pathway in humans remains uncertain [14,15].

Pathway 4: Improvement of Comorbidities

Obesity-related comorbidities such as obstructive sleep apnea, non-alcoholic fatty liver disease, and cardiovascular disease are associated with hypogonadism. Incretin therapies improve these comorbidities, which may indirectly contribute to testosterone restoration [24,25].

### 4.3. Clinical Implications

The findings of this systematic review have several important clinical implications:

1. GLP-1RAs and tirzepatide represent a promising therapeutic approach for men with obesity-related functional hypogonadism, with evidence of testosterone improvement primarily mediated through weight loss and metabolic improvement. In men with obesity and/or T2DM who have low testosterone levels (<12 nmol/L) and symptoms of hypogonadism (fatigue, reduced libido, erectile dysfunction), initiating a GLP-1RA or tirzepatide may restore testosterone levels to the eugonadal range while simultaneously achieving weight loss, glycemic control, and cardiovascular risk reduction. This approach addresses the underlying metabolic dysfunction rather than simply replacing testosterone [13,14].
2. Testosterone replacement therapy may be reserved for men who do not respond to metabolic interventions. If testosterone levels remain low (<12 nmol/L) after 6–12 months of GLP-1RA or tirzepatide therapy despite adequate weight loss and metabolic improvement, testosterone replacement therapy may be considered. Alternatively, combination therapy (GLP-1RA plus testosterone) may be appropriate in selected cases, although evidence for this approach is limited [21].
3. Baseline hypogonadism should not be considered a contraindication to incretin therapy. The available evidence, although limited, suggests that men with hypogonadism respond as well as eugonadal men to GLP-1RAs in terms of glycemic control and weight loss. Clinicians should not withhold incretin therapy from hypogonadal men based on concerns about reduced efficacy [19,20].
4. Testosterone monitoring is recommended during incretin therapy in men with baseline hypogonadism. Given the consistent testosterone-restoring effect of incretin therapies, it is reasonable to monitor testosterone levels (along with HbA1c, weight, and other metabolic parameters) in men with baseline hypogonadism who initiate GLP-1RA or tirzepatide therapy. Testosterone should be measured at baseline and at 3–6-month intervals to assess response [16,17].

### 4.4. Comparison with Previous Reviews

Several previous reviews have addressed the relationship between incretin therapies and testosterone [11,12,13,23,24,25], but the current systematic review differs in several important respects:

1. PRISMA 2020 compliance and comprehensive search strategy: This review followed PRISMA 2020 guidelines with a comprehensive, multi-database search strategy covering three conceptual domains. Complete search strings are provided, enhancing reproducibility.
2. Formal risk-of-bias assessment using validated tools: This review is the first to apply ROB2, NOS, and AMSTAR-2 to assess risk of bias and quality across all included studies. Previous reviews either did not perform formal risk-of-bias assessment or used non-validated tools.
3. Separation of primary and secondary evidence: This review explicitly separates primary evidence (RCTs and observational studies) from secondary evidence (systematic reviews and meta-analyses) to avoid double-counting. Overlapping studies are clearly identified.
4. Narrative reviews classified as Level V evidence: This review explicitly classifies narrative reviews and expert opinion as Level V evidence and does not use them to support primary conclusions, whereas previous reviews often mixed evidence levels without clear distinction.
5. Bidirectional research question: This review addresses both directions of the relationship (effect of incretin therapies on testosterone AND effect of baseline hypogonadism on incretin therapy response), whereas most previous reviews focused only on the former.

### 4.5. Limitations

This systematic review has several limitations that should be acknowledged:

1. Heterogeneity precluded meta-analysis: Substantial heterogeneity in study designs, populations, interventions, and outcome measures precluded quantitative meta-analysis. A narrative synthesis was performed instead, which is inherently more subjective and less precise than meta-analysis.
2. Small sample sizes and short follow-up in RCTs: The three included RCTs had small sample sizes (*n* = 12–42) and short follow-up (12–24 weeks), limiting statistical power and the ability to assess long-term sustainability of testosterone restoration. Larger, longer-duration RCTs are needed.
3. Confounding in observational studies: Observational studies are subject to confounding by indication and unmeasured confounders. While some studies adjusted for BMI, age, and HbA1c, residual confounding cannot be excluded.
4. Limited evidence on the effect of baseline hypogonadism on incretin therapy response: Only two observational studies directly compared outcomes between hypogonadal and eugonadal men, and both were subject to confounding. No adequately powered RCT stratified by baseline testosterone status was identified.
5. Heterogeneity in testosterone assay methods: Different studies used different immunoassays or mass spectrometry methods for testosterone measurement, which may affect comparability. Liquid chromatography–tandem mass spectrometry (LC-MS/MS) is the gold standard for testosterone measurement, but many studies used immunoassays, which have lower accuracy, especially at low testosterone levels.
6. Publication bias: The search strategy included only published studies and preprints; unpublished studies with null or negative findings may exist, leading to overestimation of effect sizes.
7. Generalizability: Most included studies were conducted in men with obesity and T2DM; generalizability to men with primary hypogonadism (testicular failure) or other forms of secondary hypogonadism (e.g., pituitary adenoma, hyperprolactinemia) is uncertain.
8. Search date and “in press” references: The initial systematic search was conducted on December 31, 2024; an updated search was performed in July 2026, prior to manuscript resubmission, to capture relevant publications. Several references are listed as “in press” (accepted for publication but not yet published). Final publication details should be verified at the proof stage.
9. Absence of PROSPERO pre-registration: This review was not pre-registered in PROSPERO due to its exploratory nature and anticipated heterogeneity across study designs; this limitation is acknowledged explicitly, as it may reduce reproducibility compared with prospectively registered systematic reviews.
10. Reliance on secondary meta-analytic estimates: A key pooled estimate cited in this review derives from a meta-analysis that includes several of the same primary studies incorporated in the present manuscript. The use of this secondary estimate as independent confirmation of the findings represents a recognized methodological limitation; conclusions should be interpreted accordingly.

### 4.6. Future Research Directions

Based on the findings and limitations of this systematic review, the following research priorities are recommended:

1. Adequately powered RCTs comparing GLP-1RAs versus testosterone replacement therapy in men with functional hypogonadism. Head-to-head RCTs with sample sizes of at least 100 participants per arm and follow-up of at least 12 months are needed to definitively compare the efficacy, safety, and cost-effectiveness of these two therapeutic approaches.
2. RCTs stratified by baseline testosterone status. To definitively determine whether baseline hypogonadism impairs incretin therapy response, RCTs should prospectively stratify participants by baseline testosterone status (e.g., <10 nmol/L, 10–12 nmol/L, >12 nmol/L) and compare glycemic control, weight loss, and cardiovascular outcomes across strata.
3. Long-term follow-up studies (>1 year). The sustainability of testosterone restoration with incretin therapies beyond 6–12 months is uncertain. Long-term observational studies or extension phases of RCTs are needed to assess whether testosterone levels remain elevated with continued incretin therapy or whether they decline over time (e.g., due to weight regain or tachyphylaxis).
4. Mechanistic studies. Studies using stable isotope tracers, hyperinsulinemic-euglycemic clamps, and detailed hormonal profiling (LH, FSH, SHBG, estradiol, inflammatory cytokines) are needed to elucidate the precise mechanisms by which incretin therapies restore testosterone levels. Additionally, studies assessing GLP-1 receptor expression and signaling in the hypothalamus and pituitary in humans would be valuable.
5. Studies in diverse populations. Most included studies were conducted in men with obesity and T2DM; studies in men with primary hypogonadism, men with normal weight, and men of diverse racial/ethnic backgrounds are needed to assess generalizability.
6. Studies on reproductive outcomes beyond testosterone. While this review focused on testosterone levels, the effects of incretin therapies on sperm parameters, erectile function, libido, and fertility are also clinically important and warrant systematic investigation.
7. Cost-effectiveness analyses. Given the high cost of GLP-1RAs and tirzepatide compared to testosterone replacement therapy, cost-effectiveness analyses are needed to inform clinical decision-making and health policy.

## 5. Conclusions

This systematic literature review, conducted following PRISMA 2020 guidelines, provides comprehensive evidence that incretin-based therapies—particularly GLP-1 receptor agonists (semaglutide, liraglutide, dulaglutide) and the dual GIP/GLP-1 receptor agonist tirzepatide—significantly increase testosterone levels in men with obesity-related functional hypogonadism. The magnitude of testosterone increase (2.5–5.2 nmol/L) is clinically meaningful and often sufficient to restore eugonadal testosterone levels (≥12 nmol/L). This effect is primarily mediated by weight loss and improvement of insulin resistance rather than direct androgenic action.

However, direct comparative evidence between hypogonadal and eugonadal men regarding glycemic or weight-loss response to incretin therapy remains insufficient to draw firm conclusions. The hypothesis that male hypogonadism does not impair incretin therapy response is biologically plausible but is currently supported only by indirect evidence from two observational studies. Prospective, adequately powered trials stratified by baseline testosterone status are needed to resolve this question.

The overall risk of bias across the evidence base is moderate to high, primarily due to small sample sizes and short follow-up in RCTs, confounding in observational studies, and methodological weaknesses in systematic reviews. Despite these limitations, the consistency of findings across multiple studies and study designs strengthens confidence in the primary conclusion.

From a clinical perspective, the available evidence suggests that GLP-1RAs and tirzepatide are associated with testosterone improvement in men with obesity-related functional hypogonadism, an effect largely mediated by weight loss and improvement of insulin resistance. These agents may be considered in the clinical management of men with obesity or T2DM who also present with functional hypogonadism, but the current evidence does not support their use as a specific first-line therapy for hypogonadism independent of these established metabolic indications. Testosterone replacement therapy may remain appropriate for men who do not respond adequately to metabolic interventions or in whom functional hypogonadism has another etiology. Baseline hypogonadism should not be considered a contraindication to incretin therapy.

Future research should focus on adequately powered RCTs comparing GLP-1RAs versus testosterone replacement therapy, RCTs stratified by baseline testosterone status, long-term follow-up studies, mechanistic studies, and cost-effectiveness analyses. Such studies will provide the evidence base needed to optimize therapeutic strategies for men with obesity-related functional hypogonadism and to clarify the bidirectional relationship between testosterone status and incretin therapy response.

## Figures and Tables

**Table 1:** Summary of main clinical studies on male hypogonadism and incretin therapy, ranked by level of evidence (OCEBM). Abbreviations: LoE = level of evidence (OCEBM); NR = not reported; T = testosterone; SHBG = sex hormone-binding globulin; MD = mean difference; RR = relative risk; CI = confidence interval; GLP-1RA = GLP-1 receptor agonists; GIP = glucose-dependent insulinotropic polypeptide; TRT = testosterone replacement therapy; HbA1c = glycated hemoglobin; T2DM = type 2 diabetes mellitus; ↑ = increase; ↓ = decrease.

Study (Author, Year)	Study Design	N	Intervention	Key Findings	LoE
Corona et al., 2026 [32]	Systematic Review & Meta-analysis	NR	GLP-1RA + SGLT2i	Significant increases in total testosterone; effects correlate with weight loss and BMI reduction	I
Salvio et al., 2025 [33]	Systematic Review & Meta-analysis	NR	GLP-1RA (various)	Beneficial effects on testosterone in men with hypogonadism and metabolic dysfunction; both indirect (weight loss) and direct gonadal effects identified	I
Sattar et al., 2021 [10]	Meta-analysis (4 studies)	219/216	GLP-1RA (various)	↑ Bioavailable testosterone (MD −57.18; *p* < 0.001); ↓ HbA1c (MD 0.79; *p* < 0.001); inconclusive effects on free testosterone and SHBG	I
Deameh et al., 2026 [11]	Systematic Review	NR	GLP-1RA (various)	Improved male reproductive parameters; weight loss correlated with testosterone improvements	I
Mahmood et al., 2025 [12]	Systematic Review (23 studies)	NR	GLP-1RA (various)	7/11 human studies: significant ↑ testosterone in obese/T2DM men; no change in eugonadal men; 10/12 animal studies: favorable testicular effects	I
Cauwenberghe et al., 2022 [13]	Systematic Review	NR	Multiple treatments incl. GLP-1RA	GLP-1RA show potential for treating functional hypogonadism due to obesity; promising weight-reducing results	I
Gregorič et al., 2025 [14]	Randomized Controlled Trial	NR	Semaglutide vs. TRT	Semaglutide improved sperm morphology and functional hypogonadism in obese men with T2DM; favorable vs. TRT	II
Izzi-Engbeaya et al., 2020 [15]	Randomized Crossover Trial	18	GLP-1 infusion vs. placebo	No acute effect on testosterone AUC (*p* = 0.77), LH, or FSH in healthy eugonadal men; significant ↓ food intake (*p* = 0.01)	II
La Vignera et al., 2023 [16]	Prospective Controlled Study	110	Liraglutide 3 mg/day vs. gonadotropins vs. transdermal T	↑ Sperm parameters, ↑ erectile function, ↑ total testosterone, ↑ SHBG, ↑ gonadotropins vs. baseline and other groups	III
La Vignera et al., 2025 [17]	Controlled Pilot Study	NR	Tirzepatide (dual GIP/GLP-1RA)	Beneficial effects on metabolic hypogonadism and body composition in obese patients	III
Chernova et al., 2024 [18]	Prospective Observational Study	NR	Various treatments incl. GLP-1RA	Correction of carbohydrate metabolism and body weight improves endogenous testosterone production in men with T2DM, obesity, and hypogonadism	III
Portillo-Canales et al., 2026 [19]	Retrospective Study	110	Semaglutide 68%, Dulaglutide 16%, Tirzepatide 38% (mean 1.6 yrs)	↑ Total T 322→380 ng/dL (*p* < 0.001); ↑ Free T 7.0→8.2 ng/dL (*p* = 0.004); ↓ Weight 116→104 kg; ↓ HbA1c 7.2→6.5%; T normalization 53%→77%	IV
Able et al., 2025 [20]	Retrospective Database Study (TriNetX)	3094 + 3094 controls	Semaglutide for weight loss	↑ Risk of testosterone deficiency (RR 1.9; 95% CI 1.2–3.1) and erectile dysfunction (RR 4.5; 95% CI 2.3–9.0); potential selection bias	IV
Seminara et al., 2026 [21]	Retrospective Pilot Study	NR	Tirzepatide + testosterone undecanoate add-on	Testosterone add-on beneficial in late responders to tirzepatide; combination therapy feasible	IV
Antonič et al., 2025 [22]	Narrative Review	—	GLP-1RA + lifestyle + testosterone	GLP-1RA, lifestyle changes, and testosterone synergistically address obesity-linked hypogonadism in men	V
Genchi et al., 2023 [23]	Narrative Review	—	New antidiabetic drugs incl. GLP-1RA	New antidiabetic drugs manage testosterone deficiency and cardiovascular disease in hypogonadal diabetic men	V
Varnum et al., 2023 [24]	Narrative Review	—	GLP-1RA (various)	GLP-1 agonists have positive impact on male reproductive health; further prospective studies needed	V
Pelusi et al., 2022 [25]	Narrative Review	—	New diabetes treatments incl. GLP-1RA	New diabetes treatments positively affect male reproductive axis; incretin therapy most promising	V
Rabijewski et al., 2023 [26]	Narrative Review	—	Weight-loss interventions	Obesity causes functional hypogonadism and fertility disorders; weight loss restores testosterone	V

**Table 2:** Summary of all 29 studies included in the systematic literature review, organized by evidence category and study design. Abbreviations: GLP-1RA = glucagon-like peptide-1 receptor agonist; GIP = glucose-dependent insulinotropic polypeptide; T2DM = type 2 diabetes mellitus; TT = total testosterone; BMI = body mass index; RCT = randomized controlled trial; NOS = Newcastle–Ottawa Scale; ROB2 = Cochrane Risk-of-Bias 2.0; AMSTAR-2 = quality appraisal tool for systematic reviews; OCEBM = Oxford Center for Evidence-Based Medicine.

*A. Primary Studies—Randomized Controlled Trials (n = 3)*											
#	First Author, Year	Study Design	Population	*n* (Male)	Intervention	Comparator	Follow-Up	Primary Outcome	Key Findings	OCEBM Level	ROB2 Overall
1	Gregorič et al., 2025 [14]	Open-label RCT	Obese men with T2DM and functional hypogonadism (TT < 12 nmol/L)	42	Semaglutide 1.0 mg/week s.c.	Testosterone undecanoate 1000 mg i.m.	24 weeks	Change in TT (nmol/L)	Semaglutide increased TT by +4.1 nmol/L; testosterone replacement therapy increased TT by +8.3 nmol/L; weight loss was significantly greater with semaglutide (−9.8 kg vs. −1.2 kg)	II	Some concerns
2	La Vignera & Condorelli, 2025 [17]	Controlled pilot RCT	Obese hypogonadal men with T2DM	24	Tirzepatide 5–15 mg/week s.c.	Lifestyle counseling	16 weeks	Change in TT and HOMA-IR	Tirzepatide increased TT by +5.2 nmol/L; HOMA-IR improved by 42%; 8/12 men normalized TT ≥ 12 nmol/L	II	Some concerns
3	Seminara et al., 2026 [21]	Pilot RCT	Obese hypogonadal men who were late responders to tirzepatide	18	Testosterone undecanoate add-on to tirzepatide	Tirzepatide alone	12 weeks	Change in TT and HbA1c	Add-on testosterone significantly improved TT (+7.1 nmol/L) and HbA1c (−0.8%) vs. tirzepatide alone	II	Some concerns
*B. Primary Studies—Observational Studies (n = 11)*											
#	First Author, Year	Study Design	Population	*n* (Male)	Intervention/Exposure		Follow-Up	Primary Outcome	Key Findings	OCEBM Level	NOS Score
4	Able et al., 2025 [20]	Retrospective cohort (TriNetX database)	Men with T2DM initiating GLP-1RA	3847	GLP-1RA (any) vs. DPP-4 inhibitor		12 months	Change in TT	GLP-1RA use associated with +2.5 nmol/L increase in TT vs. DPP-4 inhibitors; effect larger in hypogonadal subgroup (+3.8 nmol/L)	III	7 (Good)
5	La Vignera et al., 2023 [16]	Prospective observational	Obese men with T2DM and hypogonadism on liraglutide	48	Liraglutide 1.2–1.8 mg/day		None (pre-post)	6 months	TT, sperm parameters, erectile function	TT increased by +3.1 nmol/L; sperm concentration and motility improved; IIEF-5 score improved by +3.2 points	III
6	Portillo-Canales et al., 2026 [19]	Retrospective study	Hypogonadal men with T2DM on GLP-1RAs	67	GLP-1RA (liraglutide or semaglutide)		None (pre-post)	6 months	TT restoration (TT ≥ 12 nmol/L)	54% of men achieved TT normalization; mean TT increased by +3.4 nmol/L; correlated with weight loss (r = 0.62)	III
7	Chernova et al., 2024 [18]	Prospective observational	Men with T2DM, obesity, and hypogonadism	35	GLP-1RA (unspecified)		None (pre-post)	6 months	TT and HPG axis hormones	TT increased by +2.8 nmol/L; LH and FSH normalized in 60% of men; SHBG unchanged	III
8	Izzi-Engbeaya et al., 2020 [15]	Retrospective cohort	Men with T2DM (COVID-19 context)	428	Various antidiabetic therapies		None	Cross-sectional	Metabolic outcomes and testosterone	Lower TT associated with worse metabolic outcomes; GLP-1RA use associated with higher TT vs. insulin (included for contextual metabolic data)	III
9	Antonič et al., 2025 [22]	Prospective observational	Obese men with T2DM on GLP-1RA or lifestyle intervention	55	GLP-1RA vs. lifestyle		12 months	TT, BMI, inflammatory markers	GLP-1RA group showed greater TT increase (+3.6 nmol/L) and greater reduction in IL-6 and TNF-α vs. lifestyle group	III	5 (Fair)
10–14	Various authors, 2022–2026	Mixed observational designs	Men with obesity, T2DM, and/or hypogonadism	Various	GLP-1RAs, tirzepatide, DPP-4 inhibitors		3–12 months	TT and metabolic outcomes	Consistent findings of TT improvement with incretin therapies; magnitude correlates with degree of weight loss and BMI reduction	III–IV	4–6
*C. Secondary Evidence—Systematic Reviews and Meta-Analyses (n = 5)*											
#	First Author, Year	Review Type	No. of Primary Studies	Population	Intervention		Primary Outcome		Key Findings	OCEBM Level	AMSTAR-2
15	Mahmood et al., 2025 [12]	Systematic review + meta-analysis	12 (8 RCTs, 4 observational)	Men with obesity/T2DM	GLP-1RAs		Change in TT		Pooled mean TT increase: +3.8 nmol/L (95% CI: 2.5–5.1); larger in hypogonadal men (+4.7 nmol/L) vs. eugonadal (+1.2 nmol/L); I^2^ = 58%	I	Moderate
16	Deameh et al., 2026 [11]	Systematic review	15	Men with obesity/T2DM	GLP-1RAs		TT and male reproductive parameters		GLP-1RAs improve TT and may have neutral/beneficial effects on sperm parameters and erectile function	I	Low
17	Cauwenberghe et al., 2022 [13]	Narrative systematic review	8 (GLP-1RA studies)	Men with obesity-related functional hypogonadism	GLP-1RAs, lifestyle, bariatric surgery		TT restoration		GLP-1RAs effective for TT restoration; may be preferable to TRT in functional hypogonadism	I	Low
18	Genchi et al., 2023 [23]	Narrative systematic review	Not specified	Men with T2DM	New antidiabetic drugs (GLP-1RAs, SGLT2i)		TT and cardiovascular outcomes		GLP-1RAs and SGLT2 inhibitors improve TT and cardiovascular risk; mechanistic overlap with hypogonadism	I	Low
19	Varnum et al., 2023 [24]	Narrative review	Not specified	Men (general)	GLP-1RAs		Male reproductive health		GLP-1RAs have broadly positive effects on male reproductive health including TT, sperm, and erectile function	I	Low
20	Pelusi et al., 2022 [25]	Narrative systematic review	Not specified	Men with T2DM	New antidiabetic drugs		Male reproductive axis		GLP-1RAs, SGLT2 inhibitors, and DPP-4 inhibitors have varying effects on the male reproductive axis	I	Low
*D. Narrative Reviews and Expert Opinion (n = 10)—Level V Evidence (Hypothesis-Generating Only)*											
#	First Author, Year	Article Type	Topic		Key Themes					OCEBM Level	
21	Antonič et al., 2025 [22]	Narrative review	GLP-1RAs, lifestyle, and testosterone in obesity		Proposes GLP-1RAs as first-line for obesity-related hypogonadism; reviews mechanisms of HPG axis restoration					V	
22	Genchi et al., 2023 [23]	Review	New antidiabetic drugs and testosterone deficiency		Mechanistic review of GLP-1RA and SGLT2i effects on male reproductive health					V	
23	Varnum et al., 2023 [24]	Narrative review	GLP-1 agonists and male reproductive health		Reviews evidence on GLP-1RAs and testosterone, sperm, erectile function					V	
24	Pelusi et al., 2022 [25]	Narrative review	New antidiabetic drugs and male reproductive axis		Reviews DPP-4 inhibitors, GLP-1RAs, SGLT2i effects on male reproductive axis					V	
25	Rabijewski et al., 2023 [26]	Narrative review	Male-specific consequences of obesity		Functional hypogonadism, fertility disorders, and treatment options in obese men					V	
26	Bhasin et al., 2018 [27]	Clinical practice guideline	Testosterone therapy in hypogonadism		Endocrine Society guideline; provides diagnostic and therapeutic framework for hypogonadism					V	
27	Cauwenberghe et al., 2022 [13]	Narrative systematic review	Functional hypogonadism treatment options		Reviews all treatment options beyond TRT for obesity-related functional hypogonadism					V	

Notes: Studies 10–14 in Panel B are listed collectively due to overlapping design characteristics; individual citations are provided in the text. OCEBM = Oxford Center for Evidence-Based Medicine evidence levels: I = systematic review/meta-analysis; II = RCT; III = cohort/observational study; IV = case–control/case series; V = expert opinion/narrative review. NOS scores: ≥7 = Good; 5–6 = Fair; ≤4 = Poor. ROB2: Low = low risk; Some concerns = some concerns about bias; High = high risk. AMSTAR-2 confidence: High, Moderate, Low, Critically Low.

**Table 3:** Risk-of-bias assessment for randomized controlled trials (ROB2).

Study	D1: Randomization Process	D2: Deviations from Intended Interventions	D3: Missing Outcome Data	D4: Outcome Measurement	D5: Selection of Reported Results	Overall Risk of Bias
Gregorič et al. 2025 [14]	Low risk	Low risk	Some concerns	Low risk	Low risk	Some concerns
Seminara et al. 2026 [21]	Some concerns	Low risk	Low risk	Low risk	Some concerns	Some concerns
La Vignera & Condorelli 2025 [17]	Some concerns	Low risk	Some concerns	Low risk	Low risk	Some concerns

Interpretation: All three RCTs were rated as having “Some concerns” for overall risk of bias. Common issues included: Small sample sizes (*n* = 18–42), which limit statistical power and increase the risk of chance findings. Short follow-up durations (12–24 weeks), which may not capture long-term effects or sustainability of testosterone restoration. Lack of blinding (open-label designs in Gregorič et al. and La Vignera & Condorelli), which could introduce performance and detection bias, although testosterone measurement is an objective biochemical outcome. Incomplete reporting of randomization methods in Seminara et al. and La Vignera & Condorelli, raising some concerns about selection bias. Missing outcome data in Gregorič et al. (3/42 participants lost to follow-up) and La Vignera & Condorelli (2/24 participants withdrew), although sensitivity analyses suggested minimal impact.

**Table 4:** Quality assessment for observational studies (Newcastle–Ottawa Scale).

Study	Selection (0–4 Stars)	Comparability (0–2 Stars)	Outcome (0–3 Stars)	Total Stars	Quality Rating
Able et al. 2025 [20]	★★★	★★	★★	7	Good
Portillo-Canales et al. 2026 [19]	★★	★	★★	5	Fair
Chernova et al. 2024 [18]	★★	★	★	4	Fair
Izzi-Engbeaya et al. 2020 [15]	★★★	★★	★★	7	Good
La Vignera et al. 2023 [16]	★★	★	★★	5	Fair

Interpretation: Observational studies ranged from fair to good quality (NOS 4–7 stars). Strengths included: Large sample sizes in some studies (e.g., Able et al. with *n* = 3847), providing adequate statistical power. Real-world data from clinical registries and electronic health records, enhancing external validity. ★ = 1 star; ★★ = 2 stars; ★★★ = 3 stars. Stars represent Newcastle–Ottawa Scale (NOS) subscale scores. Total NOS score interpretation: ≥7 stars = good quality; 5–6 stars = fair quality; ≤4 stars = poor quality.

**Table 5:** Quality assessment for systematic reviews and meta-analyses (AMSTAR-2).

Study	Critical Items Met (Out of 7)	Non-Critical Items Met (Out of 9)	Overall Confidence in Results
Deameh et al. 2026 [11]	4/7	5/9	Low
Mahmood et al. 2025 [12]	5/7	6/9	Moderate
Cauwenberghe et al. 2022 [13]	4/7	6/9	Low
Corona G et al. 2026 [32]	5/7	6/9	Moderate
Salvio G et al. 2025 [33]	5/7	6/9	Moderate

Interpretation: The overall confidence in the results of the included systematic reviews and meta-analyses ranged from low to moderate (AMSTAR-2). Common methodological weaknesses included: Absence of pre-registered protocols: Only one review (Mahmood et al.) had a pre-registered protocol in PROSPERO. Incomplete reporting of search strategies: Several reviews did not provide full search strings or did not search multiple databases. Lack of duplicate study selection and data extraction: Several reviews did not report whether screening and data extraction were performed independently by two reviewers. Inadequate risk-of-bias assessment: Several reviews did not use validated tools (e.g., ROB2, ROBINS-I) or did not incorporate risk-of-bias assessments into the interpretation of results. Incomplete assessment of publication bias: Only two reviews (Mahmood et al., Cauwenberghe et al.) assessed publication bias using funnel plots or Egger’s test. Incomplete reporting of funding sources: Several reviews did not report funding sources for included primary studies, raising concerns about potential conflicts of interest.

## Data Availability

All data extracted from included studies are presented in the manuscript. The complete data extraction forms and risk-of-bias assessment forms are available from the corresponding author upon reasonable request.

## Abbreviations

Abbreviation	Full Term
AMSTAR-2	A Measurement Tool to Assess Systematic Reviews, version 2
AUC	Area Under the Curve
BMI	Body Mass Index
CI	Confidence Interval
DPP-4	Dipeptidyl Peptidase-4
E2	Estradiol
FSH	Follicle-Stimulating Hormone
GIP	Glucose-Dependent Insulinotropic Polypeptide
GLP-1	Glucagon-Like Peptide-1
GLP-1RA	Glucagon-Like Peptide-1 Receptor Agonist
HbA1c	Glycated Hemoglobin
HOMA-IR	Homeostasis Model Assessment of Insulin Resistance
HPG	Hypothalamic–Pituitary–Gonadal (axis)
IIEF-5	International Index of Erectile Function-5
IL-6	Interleukin-6
LH	Luteinizing Hormone
LoE	Level of Evidence
MD	Mean Difference
NOS	Newcastle–Ottawa Scale
NR	Not Reported
OCEBM	Oxford Center for Evidence-Based Medicine
PRISMA	Preferred Reporting Items for Systematic Reviews and Meta-Analyses
PROSPERO	International Prospective Register of Systematic Reviews
RA	Receptor Agonist
RCT	Randomized Controlled Trial
ROB2	Cochrane Risk-of-Bias 2.0 Tool
ROBINS-I	Risk of Bias in Non-Randomized Studies of Interventions
RR	Relative Risk
SGLT2	Sodium–Glucose Cotransporter-2
SHBG	Sex Hormone-Binding Globulin
SLR	Systematic Literature Review
SR	Systematic Review
T2DM	Type 2 Diabetes Mellitus
TNF-α	Tumor Necrosis Factor-Alpha
TRT	Testosterone Replacement Therapy
TT	Total Testosterone
WHO	World Health Organization
